# Applications of Laparoscopic Transperitoneal Surgery of the Pediatric Urinary Tract

**DOI:** 10.3389/fped.2019.00029

**Published:** 2019-02-11

**Authors:** Philipp O. Szavay

**Affiliations:** Department of Pediatric Surgery, Lucerne Children's Hospital, Lucerne, Switzerland

**Keywords:** laparoscopy, pediatric, urology, minimal, invasive, surgery, nephrectomy, pyeloplasty

## Abstract

Application of laparoscopy in pediatric urology has evolved over more than 30 years coming from a merely diagnostic use for non-palpable testes to “interventional” laparoscopy to extirpative surgery and finally to the era of reconstructive pediatric laparoscopic urology, when in 1995 Peters described the first laparoscopic pyeloplasty in a child. Laparoscopic surgery in pediatric urology became implemented increasingly in the twenty-first century with now present-day applications including the complete variety of all kind of indications for surgery for pediatric urological pathology. This article aims to provide a comprehensive overview of current indications, techniques, and outcomes of laparoscopic transperitoneal surgery of the upper as well as of the lower urinary tract for urological pathology in the pediatric patient population.

## Introduction

Application of laparoscopy in pediatric urology has evolved over more than 30 years. Beginning with merely a diagnostic use of laparoscopy for cryptorchidism in the 1980ies, indications then were broadened from “interventional” laparoscopy such as the ligation of spermatic vessels for either Fowler-Stevens procedure or varicocelectomy to extirpative surgery with the first laparoscopic nephrectomy in 1991 and the first laparoscopic partial nephrectomy in 1993, respectively. In 1995, Peters performed the first laparoscopic pyeloplasty in a child, starting the era of reconstructive pediatric laparoscopic urology, which he described still in 2004 as the ultimate challenge in this field. Progressing rather slowly in the last century, laparoscopic surgery in pediatric urology became increasingly implemented in the twenty-first century also due to achievements in available technology such as smaller instruments, cutting edge dissection and suturing devices and not to forget the use of robotic surgery. Present-day applications include the complete variety of all kind of indications for surgery for pediatric urological pathology of the upper as well as of the lower urinary tract. Some of these laparoscopic procedures are meanwhile considered as the gold standard of surgical care in the field of pediatric urology as they could proof to be comparable to if not better than conventional open surgery in terms of functional outcome along with less morbidity due to minimal invasive access. This review should give a comprehensive overview to current indications, techniques, and outcomes of laparoscopic surgery focusing on laparoscopic transperitoneal surgery on the upper and lower urinary tract, respectively, in pediatric patients.

The laparoscopic transperitoneal approach has become a multi-used, standardized approach for a large spectrum of indications in pediatric surgery and pediatric urology, respectively. It offers a maximum capacity of working space (more than retroperitoneoscopy) and therefore is suitable for all age and weight groups in the pediatric patient population, ranging from the newborn to the adolescent. It provides excellent overview, detailed visualization and augmentation which make laparoscopy the superior approach—particularly for complex anatomy and pathology, respectively (more than open surgery). Laparoscopy at present day and its current use in terms of “mini-laparoscopy,” using smaller instruments and respective ports is truly minimally invasive. This does not only result in a superior cosmesis and thus the achievement of a nearly no-scar surgery, but also proved to provide advantages with regard to less postoperative pain, shortened hospital stay and faster recovery to normal activities. The question of why to perform a procedure laparoscopically rather than open should be replaced by “why not laparoscopically.”

## Laparoscopic Surgery on the Upper Urinary Tract

### Laparoscopic Nephrectomy

Laparoscopic nephrectomy has been described as a surgical first by Koyle et al. (1) Since that time indications have been decreasing for benign disease such as multicystic dysplastic kidney disease (MDKD) in terms of more restriction for removal, while indications for malignant tumors, particularly nephroblastoma (Wilms' tumor) are not only increasing but becoming legitimized through the corresponding treatment protocols.

Laparoscopic transperitoneal nephrectomy is approached through a standard 3-trocar access to the abdomen, with one 5 mm trocar at the umbilicus as for a 5 mm-scope, as well as 2 3 (2) mm-working ports in the upper and lower abdomen of the affected side, respectively. With regard to pediatric applications in a wide spectrum of available instruments and ports ranging from 2 to 12 mm the 3 mm-instrumentation proved to be the best compromise when it comes to minimal diameter along with maximal rigidity. As in general triangulation should be the goal with respect to kidney to be removed. Surgical steps include exposure of the affected kidney, either through a retro-colonic or a trans-mesocolic access to Gerota's fascia. Further dissection should focus on exposure of the renal pedicle as safe vascular isolation will be the primary goal. A dissection of the kidney out of the surrounding tissue prior to vascular isolation is not recommended due to loss of any stability of the kidney itself and consecutive difficulties to proper exposure of the vessels. Transabdominal hitching sutures may help to lift up and thus stabilize and expose the kidney, respectively. For vessel dissection and ligation different techniques are available as well as appropriate depending on size and diameter of the vessels such as monopolar cautery, harmonic devices, or most preferably vessel sealing instruments nowadays also available as 3 mm instrumentation. This may avoid the use of larger trocars for 5 mm-instruments such as clip appliers which are not available in smaller diameters so far. Ligation of both renal artery and vein must be safe, therefore suture ligation, clips, or again vessel sealing are appropriate. In any case the surgeon should be aware of additional either arterial or venous braches supplying the kidney which have to be taken down accordingly. Care should also be taken when dissecting the renal pedicle in order not to compromise the adrenal vein on the right side and for preservation of the adrenal gland in general in case the nephrectomy is not indicated for malignancy. After careful and complete vascular isolation the kidney then is freed from its surrounding tissue to complete. Further dissection of the ureter down to the bladder may then be performed depending on the indication and whether a radical nephro-ureterectomy should be achieved. The ureter may then be ligated using ligation, suture ligation, clips or simply a PDS-loop. However, in case of non-refluxing ureter it may just dissected without ligation of the distal stump. Some surgeons even advocate leaving the ureteral stump open if it is not reflexive. Finally, the kidney can be removed through the umbilical access which might need to be bluntly dilated, in case of a tumor-nephrectomy the use of a collecting bag is mandatory. In a regular case drainage of the retroperitoneal site is not necessary and should not be considered in case of a malignancy anyway. Repositioning of the colon will help to adequately cover the retroperitoneum and therefore further reconstruction of the retroperitoneum and closure of the peritoneum, respectively, is not necessary. In case that surgery at the bladder level is carried out additionally along with nephrectomy the specimen can be removed through the consecutive Pfannenstiel incision. Another special condition is nephrectomy for non-functioning kidney along with the indication for clean intermittent catheterization for neurogenic bladder disease. In this case nephrectomy should preserve the ureter which then can be used as a retroperitoneal continent catheterizable channel when brought out laparoscopically in the lower abdomen. This procedure can spare the patient additional incisions and in addition is somewhat “elegant” as the continent catheterizable channel leads into the bladder physiologically. The implementation of single-site, single-trocar techniques, summarized as Laparoendoscopic Single Site (or LESS) Surgery has been shown to deliver comparable results for nephro-ureterectomy for pediatric patients. The procedure can be achieved safely and efficiently, irrespective of age and weight. However, owing to the fact that single-site ports are not available for smaller children and infants different surgical approaches have to be considered. The question whether LESS provides even less trauma than in conventional laparoscopy remains doubtful.

Laparoscopic nephrectomy has become the gold standard for kidney removal in infants and children for benign indications and increasingly also for malignancies. It has been proven to be safe, effective and associated with a low complication rate while offering reduced morbidity due to surgical trauma, superior cosmesis and fast recovery. It therefore has been replacing the indication for open nephrectomy in the pediatric patient population (3). However implications apply for tumor-nephrectomy and therefore indicating laparoscopic nephrectomy have to take those in account according to current treatment protocols. In addition for tumor nephrectomy lymph node sampling is crucial for surgical staging and guiding further treatment. Thus, nephrectomy alone is insufficient in terms of an oncological correct tumor nephrectomy. The quality of adequate lymph node sampling laparoscopically yet has to be proven.

### Laparoscopic Partial Nephrectomy

Laparoscopic partial nephrectomy for benign indication is done for resection of a poorly or non-functioning moiety of a duplex system. The incidence of ureteral duplication ~is 0.8%, however it represents the most common congenital anomaly of the urinary tract. The majority of duplex systems will not require surgical treatment if any. However, duplex systems becoming apparent with clinical symptoms such as obstruction and consecutive hydronephrosis—most likely in the upper pole and often associated with dysplasia, megaureter, and (ectopic) ureterocele, vesico-ureteral reflux (VUR)—most likely in the lower pole or incontinence due to ectopy of the (upper pole) ureter will require intervention. Currently accepted most common indications for partial nephrectomy of a non-functioning moiety are recurrent urinary tract infections (UTI), incontinence due to ureteral ectopy or VUR with consecutive hydronephrosis of a lower pole moiety. Laparoscopic and retroperitoneoscopic partial nephrectomy are widely accepted to be the gold standard while having replaced open surgical techniques.

Prior to laparoscopy a cystoscopy and subsequent stenting of the ureter which is supposed to be removed along with partial nephrectomy is recommended in order to facilitate later identification of both the ureters intraoperatively. Laparoscopic partial nephrectomy of a non-functioning moiety is carried out through a transperitoneal approach as described above for total nephrectomy. The patient is placed in a semi-supine position. Pneumoperitoneum (6–12 mmHg, depending on weight and age of the patient) is induced after positioning a 5 mm camera port in the umbilicus and again two 3 mm working ports. A 5 mm, 30° optic will provide adequate view. The kidney again is exposed through either a retrocolic or trans-mesocolic approach. Clear identification of renal vessel supply of the upper and lower moiety is key before dissecting in order to safely preserve the remaining moiety. Vascular control is mandatory before considering parenchymal dissection. For upper pole heminephrectomy care must be taken when mobilizing the upper pole ureter as it is crossing under the lower pole renal pedicle which has to be meticulously handled in order to avoid any damage to the vessels. This allows also clear differentiation between the upper pole vessels which then have to be dissected following ligation. In case of a lower pole partial nephrectomy the upper pole renal pedicle not necessarily has to be exposed however the surgeon must ensure the correct vascular supply and preservation of the remaining moiety. Vascular control is achieved with selective suture ligation, which can be facilitated using a hitching suture for better exposure of the moiety which is supposed to be resected. Other techniques for vessel ligation and dissection include clips (compromised by the necessity for a 5 mm trocar), the use of a harmonic knife, or nowadays available even in 3 mm a vessel sealing device which provides the ability of preparation and vessel sealing in one hand along with safety in terms of occlusion of the vessel. After vascular dissection a clear demarcation of the moiety to be resected is most often recognizable thus facilitating to determine the correct plane of parenchymal dissection. This may then be carried out using electrocautery or harmonic knife most preferably. Further dissection of the ureter as well as ligation and dissection may be performed as described above for total nephrectomy.

In case a reconstruction of the lower urinary tract a single-stage procedure is an option. The bladder is approached through a Pfannenstiel incision. This will allow removing the specimen easily. Resection of a corresponding ureterocele can be performed including an eventual bladder neck reconstruction and ipsilateral reimplantation of a lower pole ureter. Functional results proved to provide an excellent outcome in terms of renal function and bladder function (4). Early surgical intervention will avoid recurrent UTI and therefore prevent from renal scaring and consecutive loss of renal function.

Since Jordan and Winslow (5) reported on laparoscopic partial nephrectomy in 1993 it has increasingly gained acceptance (2, 6–8) despite that the operation is considered to be challenging and therefore offers limitations in terms of widespread among pediatric surgeons and pediatric urologists, respectively (3). However, with the advocated use of evolving hemostatic and dissecting devices that allowed to easier obtain vascular control and thus a more straight forward resection laparoscopic partial nephrectomy has gained more popularity among surgeons (3).

So far there exist no evidence whether the laparoscopic or a retroperitoneoscopic approach are of advantage for the patient. However, literature reflects a rather higher rate of conversion and a higher complication rate for retroperitoneoscopic partial nephrectomy than for the laparoscopic procedure. Esposito et al. published his results of a multicentric study including 102 patients undergoing partial nephrectomy in a 5 years period either by a laparoscopic or a retroperitoneoscopic approach (9). In his series, the overall complication rate was significantly higher for the retroperitoneoscopic group than for the laparoscopic group, respectively. In addition, the operating time for laparoscopy was significantly shorter than compared to retroperitoneoscopy. They concluded that laparoscopic partial nephrectomy seems to be faster and safer procedure and technically easier to perform in children compared to retroperitoneoscopic partial nephrectomy mainly due to a larger working space. In addition the possibility for complete ureterectomy in case of a refluxing system was considered to be an advantage along with laparoscopy. Multiple studies have been showing that in follow-up, that there is no functional loss of the remaining moiety (3, 4, 10, 11). Single site laparoscopic approaches have been frequently used for ablative surgery such as nephrectomy and partial nephrectomy in pediatric patients. They offer comparable results, however implications due to non-available port systems adequate to pediatric application may apply (12, 13). A recent published study considered the conventional laparoscopic approach the most preferable for nephrectomy and partial nephrectomy, respectively (14).

### Laparoscopic Pyeloplasty

Uretero-pelvic junction obstruction (UPJO) is the most common cause of hydronephrosis in infants and children. The gold standard in surgical care for UPJO has been open dismembered pyeloplasty through a retroperitoneal approach as described by Anderson and Hynes. When in 1995, Peters reported on the first pediatric laparoscopic pyeloplasty a new era of reconstructive laparoscopic surgery on the upper urinary tract begun (15). Meanwhile laparoscopic dismembered pyeloplasty in children has become an established technique. It offers superior visualization of the anatomy, accurate anastomotic suturing and thus precise reconstruction of the UPJ which promises good functional results. Therefore, laparoscopic transperitoneal dismembered pyeloplasty can be considered as the gold standard for surgical treatment of intrinsic UPJO. Indication for surgery is given in case of a reduced differential renal function (DRF) of the affected side below 40%, a decrease of DRF in repeated examination, such as renal scintigram or MRI, respectively, a relevant urodynamic obstruction in renal scintigram or MRI, respectively, recurrent urinary tract infection (UTI) and/or pyelonephritis, subjective patient complaints, such as flank pain, or a special anatomical condition such as horseshoe kidney along with obstruction, respectively.

The conventional approach for laparoscopic pyeloplasty is a 3-trocar access to the abdomen, with one 5 mm trocar at the umbilicus as for a 5 mm-scope, as well as 2 3 mm-working ports in the upper and lower abdomen of the affected side, respectively. As in general triangulation should be the goal with respect to the renal pelvis to operate on. Surgical steps of laparoscopic transperitoneal pyeloplasty are defined as gaining access to the affected kidney, either through a retro-colonic or a trans-mesocolic access to Gerota's fascia. Following the incision of the fascia as well as of the fatty capsule of the kidney, a blunt/sharp dissection will expose the (dilated) renal pelvis. Further dissection and transabdominal hitching sutures will help to further expose the pyelon in a kind that a defined and safe resection of the uretero-pelvic-junction (UPJ) can be performed. Following the resection of the UPJ, the ureter is incised and spatulated on his lateral aspect in order to provide a sufficient length of ureteral wall for achieving a wide side-to-side anastomosis. For dissection and preparation different techniques are available and appropriate such as monopolar cautery, harmonic devices, or vessel sealing instruments. The anastomosis can be performed with either a single interrupted technique or a running suture as well. The single-interrupted sutures will offer more safety in achieving a watertight anastomosis and may be more tissue-sparing as well. The running suture may allow a rather time-saving technique however requires constantly application of tension to the thread in order to avoid loosening which might be the cause for urinary leakage later. Meanwhile barbed sutures are available down to metric sizes of 4/0, which may facilitate performing a running suture in this setting. Otherwise braided sutures in sizes of 6/0 for infants and 5/0 for older patients are appropriate. An inverting technique of suturing is recommended to avoid any suturing material to be exposed to intraluminal as this might cause crystallization at the thread with consecutive bacterial colonization. There is some ongoing discussion whether to stent the anastomosis and what kind of stent to use. The use of a transabdominal, trans-anastomotic stent technique described by Obermayr et al. (16) allows an atraumatic technique with does not require a second general anesthesia to remove the stent compared to the use of any kind of double-J-stents. Other techniques include double-J-stents, percutaneous nephrostomy stents and others. In a regular case additional drainage will not be required. The question whether to put a stent in and if so how long those should stay remain to the preference of the surgeon as there is so far no evidence in favor for one of the mentioned methods.

Laparoscopic dismembered pyeloplasty has evolved to become the gold standard for the surgical treatment of intrinsic UPJO since a surgical first in 1995 by Peters (15). It has been proven to be safe, effective, and associated with a low complication rate with excellent functional results (17–23). Laparoscopic dismembered pyeloplasty on the same hand offers low morbidity due to reduced surgical trauma, superior cosmesis, fast recovery and quick return to daily and social activities. It has been therefore surpassed open pyeloplasty in many centers as the gold standard for surgical management of UPJO. In addition laparoscopy seems to be as safe and effective as primary pyeloplasty for redo-surgery in case of failed pyeloplasty (24, 25). For the diagnosis of hydronephrosis in association with a horseshoe kidney the laparoscopic transperitoneal approach has been demonstrated to offer superior visualization of the anatomy, thus providing excellent functional results (26, 27).

Compared to open surgery there have been implications coming along with minimal invasive approach techniques. The most remarkable one is probably the less reduction of the renal pelvis as compared to the original technique described by Anderson and Hynes. However, different authors considered a less reductive resection of the renal pelvis not to be determinative in terms of the functional result (28, 29). Whether to use running or single-interrupted sutures, respectively, remains to the preference of the surgeon. There might be some higher surgical efficiency with the running suture method (30). One striking advantage of transperitoneal laparoscopic pyeloplasty is that the approach *per se* is a standard procedure for many indications in both pediatric surgery and urology. In addition it is applicable also for children below 1 year of age. There is sufficient evidence in literature that also in infants laparoscopic dismembered pyeloplasty has been proven to be a safe procedure providing the same functional outcomes as the open approach (31–33). In comparing laparoscopic multiport pyeloplasty with single-site approaches such as the trans-umbilical approach it could be demonstrated that although the cosmetic result with the single-site approach is satisfactory, the multi-port access did affect the shape of the umbilicus, thus the cosmetic result was considered to be better (34). Multiple studies were aiming to describe differences in between open, laparoscopic and robotic pyeloplasties, respectively. All of those demonstrate that patients undergoing robotic-assisted laparoscopic pyeloplasty had a shorter hospital stay and less request of pain medication however, there could be no difference shown in the success rates for open, laparoscopic and robotic-assisted laparoscopic pyeloplasty, respectively (35–37). In conclusion and with regard to a higher cost associated with robotic pyeloplasty thus making it less available to the majority of patients laparoscopic pyeloplasty is considered to be equal effective as all other available techniques and therefore should be considered as the true technique of choice for surgical treatment of intrinsic UPJO in children and infants.

### Laparoscopic Uretero-Ureterostomy

Along with the evolvement of laparoscopic dismembered pyeloplasty as becoming a standard procedure different kind of procedures for reconstruction of upper urinary tract pathology derived from the technique of laparoscopic dismembered pyeloplasty. In 2008, Lowe et al. already reported their series on duplex anomalies and laparoscopic reconstruction for obstructed, dilated segments (11). The procedures performed included pyelo-ureterostomy for incomplete duplication and lower pole pelvi-ureteric junction obstruction and ipsilateral uretero-ureterostomy along with distal ureterectomy for obstruction in a dysplastic upper pole with uretereal ectopy. The experience made as well as the results achieved are corresponding to the own experience. Placement of trocars and surgical exposure are analogous to that for laparoscopic transperitoneal dismembered pyeloplasty and as described above. As for duplex system surgery again the cystoscopic placement of an ureteral stent prior to laparoscopy is highly recommended in order to facilitate identification of the ureters during the laparoscopic operation. Suturing techniques again can be performed analogous to those used for pyeloplasty. However, due to limited calibers of ureters suturing must be meticulous in order to achieve a patent and non-obstructing anastomosis. Those procedures must be considered as challenging in terms of the required technical level of expertise as well as in terms of the absolute request for being successful in order to preserve the differential renal function of the affected duplex system. Observational studies (10, 11) could show that laparoscopic reconstructional surgery on the upper urinary tract using techniques deriving from pyeloplasty can successfully be applied for a various spectrum of procedures, however there is a relative lack of evidence in literature due to non-existing prospective and randomized studies.

## Laparoscopic Surgery on the Lower Urinary Tract

### Laparoscopic Extravesical Ureteral Reimplantation

The most widespread laparoscopic procedure on the lower urinary tract in children is laparoscopic anti-reflux ureteral reimplantation. A first clinical experience with this laparoscopic application has been described by Janetschek et al. (38). They operated on six female patients girls for vesicoureteral reflux and recurrent urinary infections aged 6–10 years. The procedure performed was a laparoscopic ureteral reimplantation according to the well-established technique of Lich-Gregoir. When encountering mild unilateral stenosis, decompensating urinary tract obstruction as well as uncomplicated urinary tract infection they interestingly concluded that laparoscopic Lich-Gregoir antireflux procedure is a complicated operation offering no advantage compared to the conventional open operation (38). Lakshmanan and Fung re-defined the laparoscopic technique and concluded that the laparoscopic technique is comparable to open reimplantation techniques when reporting on their series of 71 children operated on for high grade VUR (39). With the evolvement and widespread of this technique too and the corresponding experience gained results obviously improved remarkedly as well as the perception of this procedure. Meanwhile the so called laparoscopic extravesical ureteral reimplantation (LEVUR) has become an accepted alternative to endoscopic treatment of vesico-uretereal reflux (VUR) in pediatric patients. However, the term reimplantation is somehow misleading as the technique used and described as Lich-Gregoir technique is not a true reimplantation but the creation of a sub-muscular path of the ureter done by extravesical dissection of the bladder detrusor muscle in order to achieve an anti-reflux mechanism. Current data in literature describe a success rate of up to 95% and a recurrence rate of VUR as low as of 4% in a patient population with VUR grade II–IV in a retrospective study (40). Authors concluded that compared to conventional open and endoscopic techniques LEVUR offers an acceptable success rate and better sustainability. A recent systematic review assessed five studies with a total of 69 LEVUR procedures performed representing a 96% success rate (41). However authors discussed that early success in terms of the anticipated anti-reflux procedure may be misleading when mid- and long-term effects and sequelae, respectively, will occur not until the 1st year after surgery. Thus, with regard to long-term outcomes in terms of preservation of differential renal function, absence of urinary tract infections and proper urinary drainage more evidence due to larger studies are warranted. Meanwhile reports on the application of laparoscopic-assisted extracorporeal ureteral tapering repair and ureteral extravesical reimplantation for primary obstructive megaureters attempt to show a success rate similar to the open procedure. However, again, larger trials and long-term follow-up are mandatory to justify this technique (42).

### Laparoscopic Appendico-Vesicostomy and Continent Catheterizable Channels

For the indication of complete bladder emptying in children with bladder voiding dysfunction such as neuropathic bladder dysfunction clean intermittent catheterization (CIC) is a viable option, preferably performed through the origine urethra. In 1980, Mitrofanoff described his technique of a continent appendico-vesicostomy for patients when transurethral CIC cannot be carried out for any reason (43). The laparoscopic approach for appendico-vesicostomy has been published by different authors already in 2004 (44, 45), however did not experience a widespread such as laparoscopic pyeloplasty so far. The surgical technique offers different options for the implantation of the appendix into the bladder by either using the anterior or the posterior wall, respectively. A different option for the placement of the appendico-cutaneostomy also applies by either using the classical Mitrofanoff-technique with the umbilicus or a rather pragmatic way by positioning the appendico-cutaneostomy into the right lower abdominal quadrant. In case of using the umbilicus care must be taken to prepare a triangular skin flap at the umbilicus up front when introducing the first trocar at the umbilicus in order to later properly implant the appendix. Two more working ports may then be used left and right of the umbilicus to provide triangulation for approaching the appendix and bladder, respectively. As in the open technique identification of the appendix and mesoappendix is followed by ligation of the appendiceal basis and the dissection of the appendix while carefully preserving the blood supply through the mesoappendix. Next the bladder wall is dissected either starting from the urachus to attempt the anterior bladder wall or by approaching the posterior bladder wall. Herefore, a transabdominal suture, hitching the bladder dome up to the ventral abdominal wall will facilitate exposure. After cystostomy the anastomosis of the appendico-vesicostomy using the distal end of the appendix is carried out by using a single interrupted suturing technique. A subsero-muscular tunnel analogous to the technique of Lich-Gregoir and described above may be used to create an anti-reflux mechanism. Filling the bladder with sterile saline will facilitate dissection and cystostomy, respectively. A third working port may be used for bringing out the appendix at the desired place either to the umbilicus or to the right lower abdominal quadrant. In the latter case a sub-peritoneal tunnel may be therefore created. Care must be taken again for preserving the blood supply, non-torsening of the appendix and the respective meso-appendix as well as for an atraumatic technique in bringing the appendix to the skin. Deflating of the CO_2_ pneumoperitoneum is mandatory in order to provide a proper alignment of the appendiceal channel. Prior to anastomosing the proximal end of the appendix to the skin ease of catherization must be proved and reproducible without obstruction until a Foley catheter is then left in place before finishing the procedure. During the check of ease of catheterization, the presence of any urinary leakage must be ruled out additionally. As an alternative to the Lich-Gregoir like anastomosis of the appendico-vesicostomy and in order to decrease surgical time and the demanding character of the procedure Weller et al. described an adaptation of the Schanfield ureteral implantation technique fixing the appendix to the anterior bladder wall using a single “U-Stitch” (46). After creation of a submucosal tunnel sharp dissection for the detrusorrhaphy follows and finally the mucosa is incised at the distal end of the submucosal tunnel. With a catheter in place a single U-stitch is performed and fastened extracorporeally with a Roeder knot in order to have an instrument available to guide the appendix into the bladder opening while the knot is then being tied. The use of an extra port may be an alternative to perform this step. The detrusor is then closed over the appendix thereby creating the antireflux mechanism. Authors concluded that this technique reduced operative time and made the procedure technically easier.

Since Hsu and Shortliffe published the first complete laparoscopic appendico-vesicostomy in 2004 (45) different authors have been reporting on both laparoscopic as well as robotic assisted appendico-vesicostomies in pediatric patients. However, operating times so far are of remarkable length ranging from more than 360 to still 180 min for a procedure when done alone while open surgery would require a much shorter operating time. With the modification described by Weller et al. (46) a significant reduction of the operating time could be achieved in a small patient series. As in the open procedure pitfalls and complications are mainly due to anastomotic leakage and persisting hematuria intra- and peri-operatively while issues with catheterization occur peri- and post-operatively but can compromise if not ruin the operative result. For laparoscopic appendico-vesicostomy limitations are to anatomy, i.e., lenght of the appendix, working space and last but not least experience of the surgeon. In terms of feasability but moreover the functional result the procedure must be considered as being highly demanding. However, in the observational studies published so far, intracorporeal laparoscopic appendico vesicostomy proved to be safe and effective offering a superior cosmesis but the same kind of potential complications as known from the open procedure (47).

Beside the laparoscopic appendico-vesicostomy other types of continent catheterizable channels or conduits, respectively, may apply to a laparoscopic approach. In the own experience the use of ureter when performed along with the indication for unilateral nephrectomy provided an elegant option for a modified Mitrofanoff-stoma, avoiding the necessity of implantation of the catheterizable channel into the bladder. Following nephrectomy and preservation of the ipsilateral ureter, the ureter is guided through a sub-peritoneal tunnel of the corresponding side of the abdominal wall before being delivered through the abdominal wall and getting anastomosised to the skin in either the left or right lower abdominal quadrant, respectively. Again ease of catheterization must be ensured.

### Laparoscopic Augmentation Cystoplasty

Bladder augmentation for surgical management of neuropathic bladder dysfunction is a procedure often performed in the context of other reconstructive procedures such as appendico-vesicostomy or bladder neck reconstruction resulting in complex reconstructive surgery individually stratified for the patient and therefore being demanding while requesting long operating times. The procedure of bladder augmentation can be performed using the (mega-) ureter when nephrectomy is anticipated on the same occasion. Auto-augmentation is another option however offers limited increase of bladder capacity and questionable functional results on the long run. At present day augmentation of the bladder using ileum—so called ileocystoplasty—represents a currently widely accepted standard of care. Ileo-cystoplasty itself requests resection and proper continuity-restoring anastomosis of the small bowel as well as of a continent anastomosis of the ileal segment to the mostly small and hypertrophic bladder thus being demanding in terms of suturing. Taking all the above mentioned into account, it may be justified to call a pure laparoscopic attempt to this operation ambitious even more when done in combination with other procedures such as appendico-vesicostomy.

Beginning in 1993, a first report on laparoscopic auto-augmentation was published by Ehrlich and Gershman (48), and followed by a first report on a laparoscopic bladder gastrointestinal augmentation using stomach in 1995 by Docimo et al. (49). At that time 5 trocars were used and stapling and suturing devices, respectively, to facilitate suturing. The operation took more than 10 h. It took 10 more years for Shadpour to be the first reporting on 5 patients in whom ileo-cystoplasty could be performed without the use of stapling devices applying complete intracorporeal suturing using a 3-port laparoscopic approach (50). Pure laparoscopic enterocystoplasty could also be demonstrated by Lorenzo et al. (51) using a 3-trocar technique and stapling devices. Authors considered it to be an advanced procedure that is technically demanding (51). With the evolvement and increasing implementation of laparoscopic robotic surgery it could be shown that laparoscopic augmentation ileo-cystoplasty along with appendico-vesicostomy (Mitrofanoff) can be done purely intracorporeally including harvesting of ileum, bowel anastomosis, and the continent anastomosis of the ileal segment to the bladder in a 5-port-technique with the use of a robot (52). At the same time hybrid procedures as being a laparoscopic assisted uretero-cystoplasty intended to reduce surgical trauma, to be less invasive as well as to offer improved cosmesis (53). A rather recent publication reported on a combined laparoscopic-assisted nephrectomy, augmentation uretero-cystoplasty and Mitrofanoff-appendico-vesicostomy, using a 3 trocar technique and a Pfannenstiel-incision to access the bladder (54).

Although all those reports provided a less invasive option for bladder augmentation, proof of evidence in terms of being equivalent or even superior to conventional open surgery is lacking so far. However, those attempts for a more minimal invasive approach to even complex reconstructive surgery in pediatric urology demonstrate the potential of laparoscopic applications in pediatric urology in general.

## Conclusion

Application of laparoscopy in pediatric urology has evolved from a diagnostic use to “interventional” laparoscopy to extirpative surgery to reconstructive pediatric laparoscopic urology. Along with the progress in the expertise of pediatric surgeons and pediatric urologists achievements made with available technology lead to a highly complex spectrum of present-day applications including a large variety of indications for surgery for pediatric urological pathology of the upper as well as of the lower urinary tract. Some of these laparoscopic procedures are meanwhile considered as the gold standard of surgical care in the field of pediatric urology as they could prove to be comparable to if not better than conventional open surgery in terms of functional outcome along with less morbidity due to minimal invasive access. Others lack this kind of evidence but may understood as an attempt for a continuous progress in making pediatric urological surgery less invasive. This would result in not only superior cosmesis but providing a true benefit for the pediatric patient in terms of less postoperative pain, shortened hospital stay and faster recovery to normal activities. The future is bright. Single site surgery as well as robotic surgery are both areas of growth and continuous development in pediatric urology that will innovate future surgical treatment options (55). The use of 3D vision, along with articulating instruments will diminish the distinction between current robotic-assisted and conventional laparoscopy (56) thus providing the best out of two worlds in a hybrid application which will evolve to become not only a true alternative to current concepts but maybe a future standard of care. There are many more applications of laparoscopy for pediatric urology such as transperitoneal laparoscopic lithotomy or laparoscopic ureteral replacement (57, 58). So again, the question should be raised why not operate on laparoscopically.

## Author Contributions

The author confirms being the sole contributor of this work and has approved it for publication.

### Conflict of Interest Statement

The author declares that the research was conducted in the absence of any commercial or financial relationships that could be construed as a potential conflict of interest.
